# Phenotyping urban built and natural environments with high-resolution satellite images and unsupervised deep learning

**DOI:** 10.1016/j.scitotenv.2023.164794

**Published:** 2023-06-13

**Authors:** A Barbara Metzler, Ricky Nathvani, Viktoriia Sharmanska, Wenjia Bai, Emily Muller, Simon Moulds, Charles Agyei-Asabere, Dina Adjei-Boadi, Elvis Kyere-Gyeabour, Jacob Doku Tetteh, George Owusu, Samuel Agyei-Mensah, Jill Baumgartner, Brian E Robinson, Raphael E Arku, Majid Ezzati

**Affiliations:** a Department of Epidemiology and Biostatistics, School of Public Health, Imperial College London, London, UK; b MRC Centre for Environment and Health, Imperial College London, London, UK; c Department of Informatics, University of Sussex, UK; d Department of Computing, Imperial College London, London, UK; e Department of Brain Sciences, Imperial College London, London, UK; f School of Geography and the Environment, University of Oxford, UK; g Regional Institute for Population Studies, University of Ghana, Accra, Ghana; h Department of Geography and Resource Development, University of Ghana, Legon, Accra, Ghana; i Institute of Statistical, Social & Economic Research, University of Ghana, Accra, Ghana; j Department of Epidemiology and Biostatistics, McGill University, Montreal, Québec, Canada; k Department of Equity, Ethics and Policy, McGill University, Montreal, Québec, Canada; l Department of Geography, McGill University, Montreal, Québec, Canada; m Department of Environmental Health Sciences, School of Public Health and Health Sciences, University of Massachusetts, Amherst, USA; n Abdul Latif Jameel Institute for Disease and Emergency Analytics, Imperial College London, London, UK

**Keywords:** urban environment, built and natural environment, high-resolution satellite images, clustering, deep learning, sub-Saharan Africa

## Abstract

Cities in the developing world are expanding rapidly, and undergoing changes to their roads, buildings, vegetation, and other land use characteristics. Timely data are needed to ensure that urban change enhances health, wellbeing and sustainability. We present and evaluate a novel unsupervised deep clustering method to classify and characterise the complex and multidimensional built and natural environments of cities into interpretable clusters using high-resolution satellite images. We applied our approach to high-resolution (0.3 meters/pixel) satellite image for Accra, Ghana, one of the fastest growing cities in sub-Saharan Africa, and contextualised the results with demographic and environmental data that were not used for clustering. We show that clusters obtained solely from images capture distinct phenotypes of the urban natural (vegetation and water) and built (building count, size, density, and orientation; length and arrangement of roads) environment, and population, either as a unique defining characteristic (e.g., bodies of water or dense vegetation) or in combination (e.g., buildings surrounded by vegetation or sparsely populated areas intermixed with roads). Clusters that were based on a single defining characteristic were robust to the spatial scale of analysis and the choice of cluster number, whereas those based on a combination of characteristics changed based on scale and number of clusters. The results demonstrate that satellite data and unsupervised deep learning provide a cost-effective, interpretable and scalable approach for real-time tracking of sustainable urban development, especially where traditional environmental and demographic data are limited and infrequent.

## 1 Introduction

More than 55 percent of the world’s population currently lives in cities and the percentage is projected to increase, particularly in the developing world^1^. The manner in which cities grow and change has major, local and global, environmental, health and wellbeing implications^2–4^. Timely information on the urban built and natural environment is needed to guide and evaluate policies for sustainable and healthy urban development. Yet, data on different features and dimensions of cities’ environment – including vegetation, roads, buildings, and population settlements – are scarce and infrequent in the developing world and, when available, have varying spatial and temporal coverage and resolutions. As a result, studies of urban environments are largely focused on individual features, such as the urban extent^5,6^, land use and landcover^7–11^ including green and blue spaces^12,13^, roads and connectivity^14–16^, and population density^17^, in isolation. These features are, however, often interrelated and exhibit complex patterns, at various scales, in terms of arrangements of different forms of vegetation, buildings of various sizes, and street networks. For example, many cities in developing countries contain high-density informal settlements that are scattered across the city, and which have a large number of small buildings, narrow unpaved roads, and low levels of vegetation. Outside the city centre, buildings may be larger and free standing, surrounded by some vegetation and connected by paved as well as unpaved roads, while on the periphery there may be farmlands and woodlands alongside emerging residential areas^18^.

Advances in machine learning and computer vision allow automated large-scale analysis of cities from images, as detailed in Section 2. These techniques, if applied to very high-resolution satellite images, enable studying the complexity of the urban environment at different scales, from submeter to entire neighbourhoods^19,20^. Among these, unsupervised algorithms have the potential to identify patterns in images that go beyond predefined criteria and labels, and hence untangle the complex multidimensional heterogeneities of cities’ environment^21^. We hypothesise that using solely the visual information captured by satellite images can provide practical information about spatial arrangement of the built and natural environment in a spatially consistent and coherent manner. The captured phenotypes can inform, and track the impacts of, urban planning and policy choices. To investigate this potential, in this paper we used unsupervised deep learning together with very high-resolution satellite images to identify interpretable phenotypes of the urban environment. We tested our approach in Accra, the capital of Ghana, one of the fastest growing cities in the developing world and tested the sensitivity of the results to key methodological choices. We also explored the intermediate outcomes of the deep learning method to understand if the neural network learns meaningful image representations and tested the interpretability of the resultant clusters with demographic and environmental data that were not used for clustering.

## 2 Data, methodological context and contributions

Remote sensing images, typically captured by satellites, are a valuable source of information for planning and making policy decisions about cities. Satellite images contain information at various spatial scales, ranging from features of the built and natural environment, such as buildings and trees, at the meter and increasingly submeter scale to surface and land use variation over hundreds of meters, and a combination thereof^22^. Some urban features studied with satellite images include roads and buildings^14–17^, the spatial extent of cities^5,6^, and land use and cover^7–11^. Traditionally, this information was derived through approaches such as spectral indices^23^ and pattern and texture extraction methods^24^. These methods have been increasingly complemented with deep learning techniques such as convolutional neural networks (CNN) that allow classification without explicit prior selection of low-level features^19,25^. Such supervised analyses require labelled data that represent ground-truth on one or multiple outcomes, such as buildings or roads. It is therefore difficult to use supervised methods for detecting complex combinations of urban characteristics because pre-defining features, their mutual relationships, and accessing labelled data for multi-dimensional outcomes is challenging.

Satellite images have also been used in an agnostic unsupervised approach that identifies patterns in images based on all visible features and without predetermined labels. The majority of unsupervised analyses of satellite images have been conducted at pixel-level, where pixels are grouped together based on their colour, intensity or measures such as Normalized Difference Vegetation Index (NDVI; an indicator of vegetation in a satellite image based on spectral absorption of light; range: -1.0 to 1.0) for land use or cover classification^24,26^, using a range of algorithms including k-means^27^ and its variant Iterative Self-Organising Data Analysis (ISODATA)^28^, or fuzzy approaches where the pixels can be assigned to multiple clusters^29^. Pixel-level analysis, however, might miss complex and context-related features of the urban environment which involve information at larger or multiple scales, such as the difference between inner-city greenery and vegetation contiguous to city boundaries. These distinctions are essential for urban policies and infrastructure whose impact goes beyond the area covered by a pixel^20^. Furthermore, as the spatial resolution of satellite sensors increases (<1 meter/pixel), the semantic meaning of an individual pixel diminishes^30^ since pixels cover distances smaller than most features of interest. Rather, in very high-resolution images groups of pixels collectively contain information about features of the urban environment. These features can be as small as cars^31^ and patches of greenery or water, and with increasing number of pixels capture more complex features such as rooftop materials^32^, gardens^13^, arrangement of buildings^33^, and road quality^15,16^ and connectivity^14^. The alternative to pixel-level analysis is patch-level analysis, where a CNN can simultaneously learn features of different levels of abstractions^34^ and cluster assignments^35,36^ for complex scenes at relevant scales for urban form, e.g., 30-100m^9,19,25^. This approach is analogous to fields such as population biology^37^ and genomics^38^, which commonly use data-driven methods to categorise study subjects in ways that single traits, or their pre-specified combinations, cannot.

Our study presents an unsupervised deep learning method that includes combined feature extraction and clustering. We apply the framework to satellite images of a city in the developing world where urban change has been heterogeneous and data are scarce^39–41^. To test our overall hypothesis, we interpret the clusters against external features from various data sources on built and natural environment and population, visually and through application of a post-hoc supervised classifier, and show that the clusters capture distinct features of the environment.

## 3 Study area

We applied and tested our unsupervised clustering approach in Accra, Ghana. Accra is one of the fastest growing cities in the developing world, with diverse environmental characteristics. We also had data on the built environment, water, vegetation, and population which could help with interpreting the results of our image-based analysis.

The Greater Accra Metropolitan Area (GAMA) is the administrative, economic and political capital of Ghana, with a population of ~5 million inhabitants^42^ and an area of ~1,500 km^2^. Foreign investment, and trade in natural resources such as oil and minerals, have made it a leading hub for trade, technology and education in Africa. GAMA includes the Accra Metropolitan Area (AMA) at the coast, the adjacent metropolis of Tema to the east and further suburban municipalities in the northeast and northwest. The population of GAMA increased by almost 90 percent from 2000 to 2021^42^. Economic and population growth has led to the development of luxury housing parallel to the expansion of informal settlements and slums, the latter of which are affected by poor housing and sanitation^43–46^. These variations and inequalities occur both within and between neighbourhoods. For example, Nima, a large slum located in the centre of AMA, is situated near the wealthy neighbourhood of Cantonments in the east, while livelihoods also vary between the inhabitants in Nima^47^. Other large informal settlements are located close to the wetlands at the coastal south-western part of the AMA^43^. As a result of expansion and land use changes, Accra’s natural environments such as forest, grassland and wetland have decreased^48^. Like other cities in the developing world, urban sprawl and increase in the number of vehicles have led to an expansion of road infrastructure in GAMA^49,50^. However the expansion has been uneven with most of the major paved roads in AMA, and many unpaved connecting roads elsewhere^50^.

## 4 Data and Methods

**Fig. 1:**
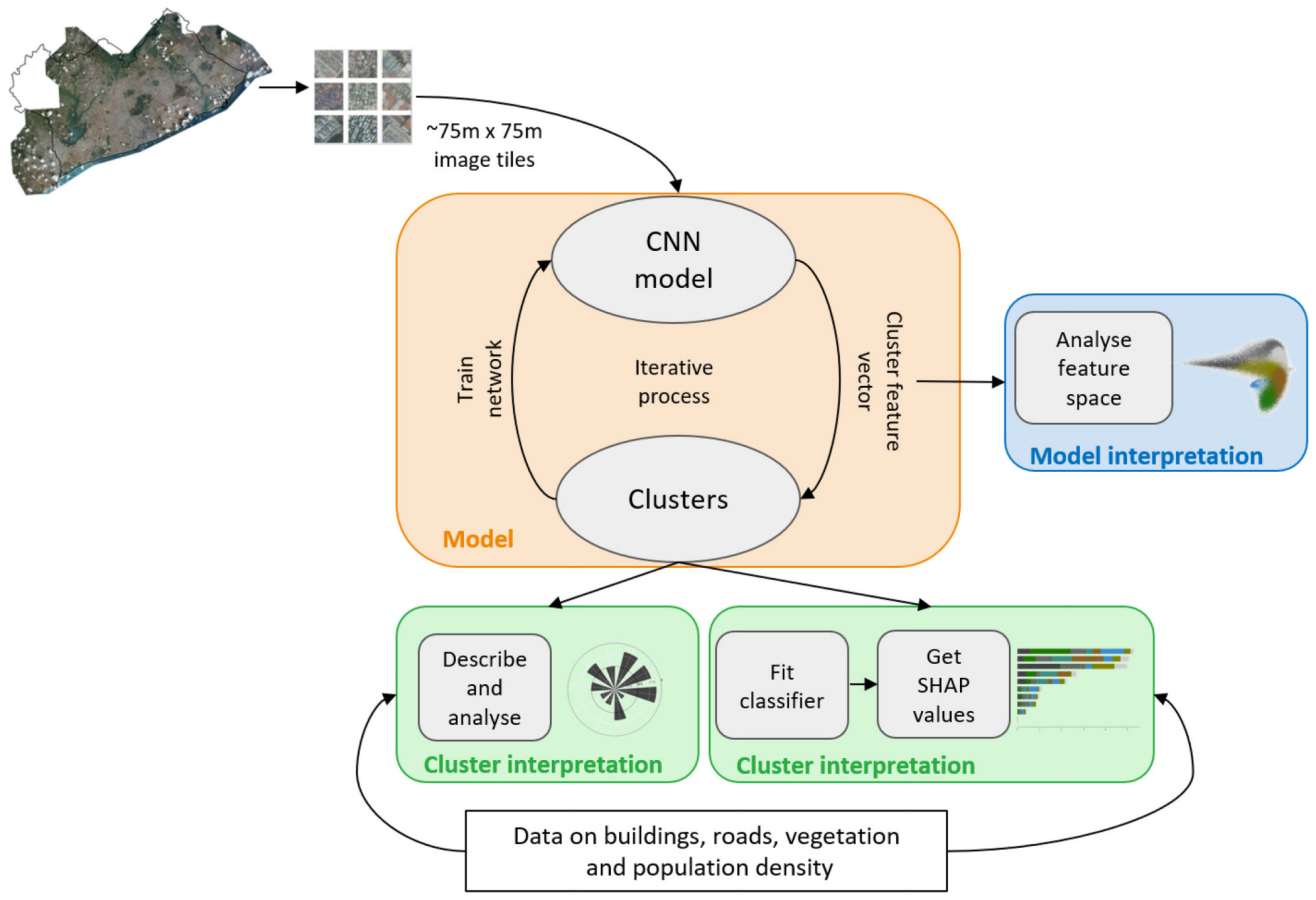
Overview of data management, analysis, and interpretation. In the pre-processing step, the city-wide satellite image was cropped into tiles. Tiles, which were not within the Greater Accra Metropolitan Area (GAMA) or contained clouds were removed. The image tiles were then fed into the end-to-end DeepCluster convolutional neural network (CNN) which is described in Methods. The resultant clusters were described and analysed with external data on buildings, roads, population density, water, and vegetation. After clustering, we fitted a classifier to predict cluster membership to understand which environmental variables were most important for cluster formation, measured by their corresponding SHAP values. To understand how the model learns the image representations, we analysed the clusters in the low-dimensional feature space.

We applied an unsupervised clustering method to a very high resolution satellite image of the city of Accra in Ghana, and interpreted the results with external data of the built and natural environment. The overall analysis plan, including data pre-processing and analysis, is shown in Fig. 1. The image and environmental data are described in Section 4.1, and the clustering method in Section 4.2, including how we used intermediate outcomes of the clustering method to evaluate the relationships among the resulting clusters. Section 4.3 describes the methods for fitting a classifier to predict cluster membership and subsequent SHAP analysis to quantify which environmental and demographic characteristics define the clusters. Additionally, we tested the sensitivity of the clustering to key methodological choices, as described in Section 4.4.

### 4.1 Data

#### 4.1.1 Satellite image

We used a very high-resolution (0.3 metres per pixel) satellite image with three different bands (RGB). The satellite image covered 87 percent of GAMA in the year 2019, missing some parts in the northwest of the region. The satellite raster data were released as part of the Maxar Open Data initiative^51^ in response to the COVID-19 pandemic in GeoTIFF format. The commercial pre-processing of the image included colour-balancing and orthorectification.

We first cut the satellite image into 256 x 256 pixel tiles, equivalent to about 75 m x 75 m on the ground. This tile size can contain multiple features of urban form, e.g., houses and roads, such that it captures objects with their urban context and surroundings. As part of a sensitivity analysis, we evaluated the influence of three different tile sizes: 128 x 128 (i.e. 50% smaller than main analysis in side length), 384 x 384 (50% larger), and 512 x 512 (100% larger) pixels. We only used tiles that were within the GAMA boundary in the analysis (Fig. 1). Additionally, tiles that contained clouds were removed from the dataset. We identified clouds by calculating the median tile colour and filtered out tiles that appeared mostly white (all RGB values > 210). The 85.4 GB citywide satellite image was divided into 321,820 256 x 256 pixel tiles, with 222,420 tiles remaining after data cleaning.

#### 4.1.2 Built environment, water, vegetation, and population

We used four different datasets on urban characteristics in Accra to interpret the image-based clusters. The datasets are described in Table 1 and mapped in GAMA in the Appendix Fig. A.1. To calculate the per tile statistics, we intersected each vector and raster dataset with the index grid of the satellite image tiles.

### 4.2 Deep Cluster: Combined feature extraction and clustering

We used DeepCluster^35^, an end-to-end feature extraction and clustering framework to assign each image tile to a cluster. In the method, a CNN is used as a feature extractor to reduce the high dimensional image to a lower dimensional feature vector. The CNN extracts visual features hierarchically, with features such as lines and edges in early layers and domain-specific features such as rooftops and trees in latter layers of the network, ultimately generating a representation that summarises each image tile into 4096 numerical features. The extracted features are reduced from 4096 to 256 with use of principal component analysis and normalised using the Euclidean norm. A k-means clustering algorithm is then applied to these features in the last layer of the CNN. The clustering algorithm assigns a cluster membership to each image representation which is then used as a pseudo-label to update the weights of the CNN classifier. The algorithm iteratively minimises clustering and classification loss, creating new pseudo-labels after each training epoch, i.e. every time the whole dataset has passed through the CNN and the weights of the network are updated. The method combines feature extraction and clustering in an end-to-end approach, meaning the cluster formation relies entirely on the image data without any use of external label data or user-guided input.

We used the CNN architecture VGG-16^52^ that has been pre-trained on the ImageNet dataset for feature extraction. CNNs that have been trained on millions of images such as the ImageNet dataset are commonly used to make use of previously learned low- and mid-level features which are similar across tasks, even if the ImageNet images are different from those used in the final analysis, hence improving and speeding up the learning process^53,54,55^. This approach has been shown to be advantageous to training from scratch especially in scenarios where the target task does not rely on labelled samples^55^. Most hyperparameters (i.e., settings for the configuration of the training process) were kept at the same values as in the original DeepCluster paper, except for the number of clusters (which is discussed in Section 4.4.1), learning rate and the number of epochs, which were set to 0.0001 and 20 respectively based on an initial set of experiments. The algorithm formed the most intuitive and interpretable clusters at a learning rate of 0.0001, compared to a set of alternative learning rates (0.1, 0.01, 0.001, 0.00001 and 0.000001). Learning rates > 0.0001 picked up structure in the city-wide satellite image that resulted from how the city-wide image was stitched together (from multiple satellite images) as part of the commercial pre-processing and was unrelated to the content of the image. The training time for around 20 epochs is about 24 hours, and training for another 24 hours (~50 epochs) did not substantially change the clusters and their interpretation.

Many cities in the developing world, including Accra, have fragmented spatial structures with pockets of slums neighbouring high-rise business buildings and scattered fringe developments^56^. To avoid smoothing over heterogeneities that can result in missing the full complexity of an urban system, we analysed the tiles independent of their proximity to other image tiles, i.e. the tiles fed into the neural network contain no information about neighbouring tiles or geographic information, e.g., latitude and longitude.

#### 4.2.1 Visualising the clusters in the feature space

To understand to what extent DeepCluster learns the intermediate image representations that distinguish clusters, we inspected the cluster membership in the lower-dimensional feature space. For this purpose, we obtained the 256 principal components of the image representations extracted by the second last fully connected layer of the CNN. These 256 principal components together accounted for 99.5% of the variance of the 4096 extracted features which the CNN uses to represent each image. We measured the degree of uniformity of tiles in the feature space that fall within each cluster with the average distance to the cluster centroid (intra-cluster distance). We show the visualisations and results of the feature space in Section 5.2.

### 4.3 Built and natural environment and demographic characteristics and predictors of clusters

We used the data on built environment, water, vegetation, and population to quantify the characteristics of the clusters that were formed based on image data alone. We report the median values of each measure for all the tiles that fall in each cluster. Additionally, we used the machine learning classifier XGBoost^57^ to quantify which environmental and demographic characteristics, individually and collectively, characterise the image-based clusters. This decision-tree-based method identifies which environmental and demographic variables predict cluster membership. It has the practical advantage of being able to accommodate missing values^58^, such as for tiles that have no buildings and therefore no average building size and orientation. To measure which environmental and demographic variables are important for predicting image tiles’ membership to different clusters, we used the fitted classifier to generate SHapley Additive exPlanations (SHAP)^59^ values. The SHAP values are summary measures of the importance of each environmental and demographic variable for each cluster as well as across all clusters, in an additive manner.

We split the dataset (tiles) into 80 percent training and 20 percent testing data. We used a stratified approach for splitting the tiles to ensure that all clusters were equally present in the evaluation. The gradient boosting classifier, XGBoost, was fine-tuned with a 5-fold cross-validation method with classification accuracy as a score. We used the Hyperopt^60^ library, which uses Bayesian optimisation for parameter tuning to find the optimal hyperparameters. The final accuracy of the classifier was 0.66, scoring 54% higher than random assignment of a given image tile to a cluster. This classification accuracy score, which is only moderate, reflects that the environmental and demographic variables that were used in the SHAP analysis are only a subset of those that have visual signals. Other visual signals, on which we did not have geocoded data, may include vehicles, building type and material, specific vegetation categories, and types of terrain^61^.

### 4.4 Sensitivity analysis

#### 4.4.1 Sensitivity to scale and number of clusters

We analysed the robustness of the clusters to scale of analysis (i.e. tile size) and the choice of number of clusters.

First, we investigated how spatial scale of analysis impacted the cluster formation. While the main analysis uses tiles sized 256 x 256 pixels, we tested a set of tiles that were smaller (128 x 128, 50% less than the main analysis) and larger in side-length (384 x 384, 50% more and 512 x 512, 100% more) than the main analysis to examine how cluster membership and characteristics changed. We report the robustness of the clusters to scale in Results (Section 5.4.1).

In the main analysis, we present eight clusters of urban environment, each with its own phenotypic characteristics. This number was chosen based on visual inspection of results, and an initial set of experiments where we sought to achieve a balance between the separation of clusters and the level of detail required for an intuitive classification of the urban environment. To further understand how the choice of cluster number influences the separation of the city into clusters and the character of the resultant clusters, we modified the number of clusters, K, in the DeepCluster analysis and report how the clusters change from K=2 to K=12 in Results (Section 5.4.2).

#### 4.4.2 Influence of hyperparameter k on feature learning

DeepCluster iteratively groups the image representations deep in the network with a standard clustering algorithm, k-means, and uses the subsequent assignments as supervision or pseudo-labels to update the weights of the network as part of a classification task. The choice of the hyperparameter k in the k-means clustering part of the algorithm is distinct from the final number of clusters (K) in the data; rather, k influences how the algorithm learns distinctive image representations. It may be the case that a large hyperparameter k is better suited for feature learning^35,62^ even if for interpretation we prefer a smaller number of clusters. To examine the role of the number of clusters in the k-means algorithm on learning image representations, we carried out the analysis as a two-step approach: in the first step, the CNN is used to create deep image representations (features) with specific values of k, and subsequent step of clustering into K clusters. To investigate whether a larger k helps to learn more discerning features, we created a set of different deep features (DF) with the three choices of k (k ∈ {8, 50, 100}), namely DF_k8_, DF_k50_, and DF_k100_. We then clustered the intermediate DF with a k-means (K=8) (i.e. as in the main analysis) to compare the cluster memberships, as reported in Section 5.4.3. Based on our stability analysis, we chose to continue the analysis with k=8, such that the last epoch cluster assignments directly mapped to final cluster labels.

## 5 Results

### 5.1 Clusters of the urban environment

**Fig. 2:**
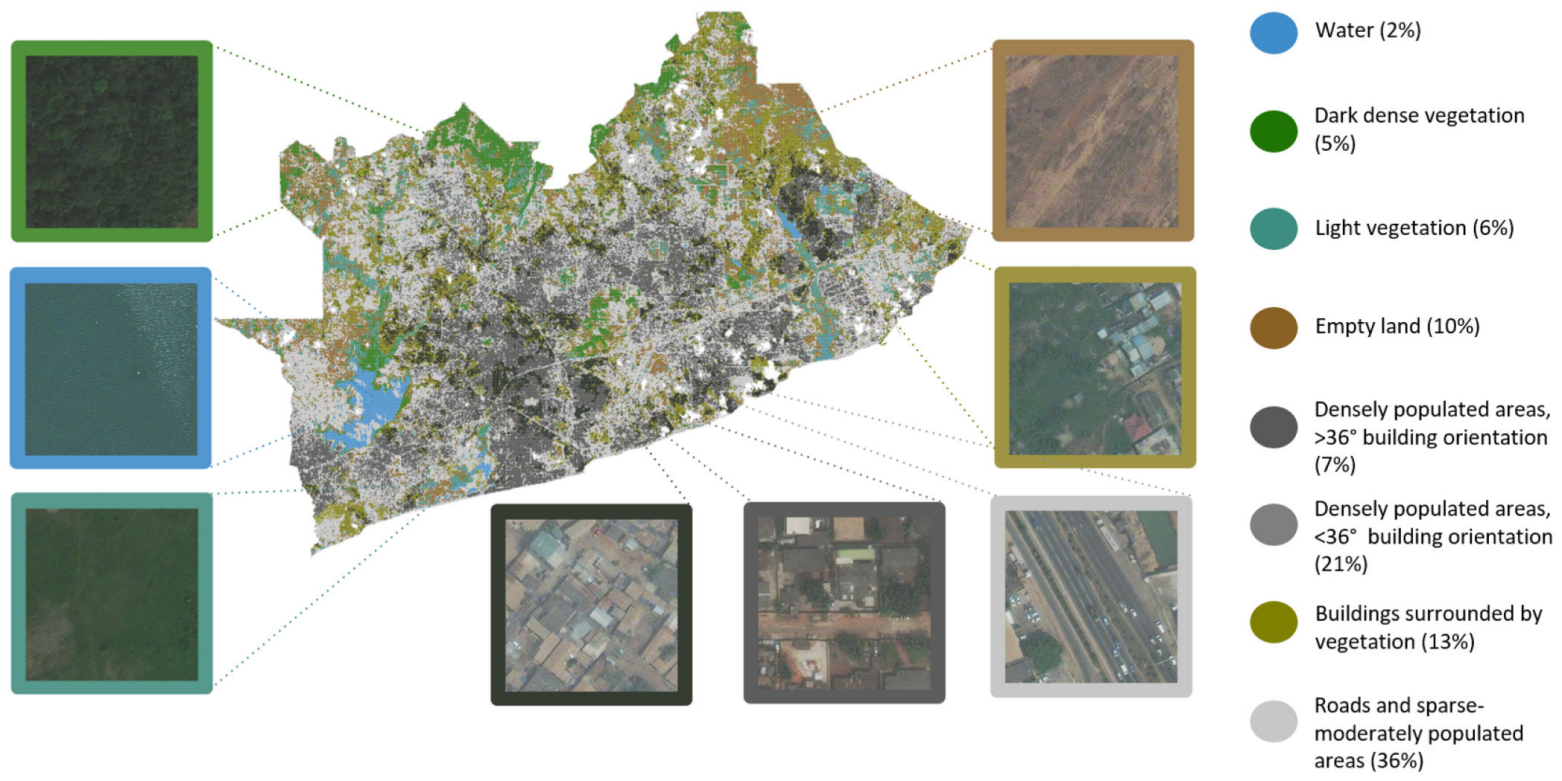
Map of image-driven clusters in the Greater Accra Metropolitan Area. The figure shows 222,420 image tiles each assigned to one of eight clusters. Each cluster is shown in a different colour – the same colour is used for each cluster in subsequent figures. The boxes show examples of the tiles that were assigned to each cluster. The numbers next to each cluster name show the percentage of tiles grouped into the cluster.

**Fig. 3:**
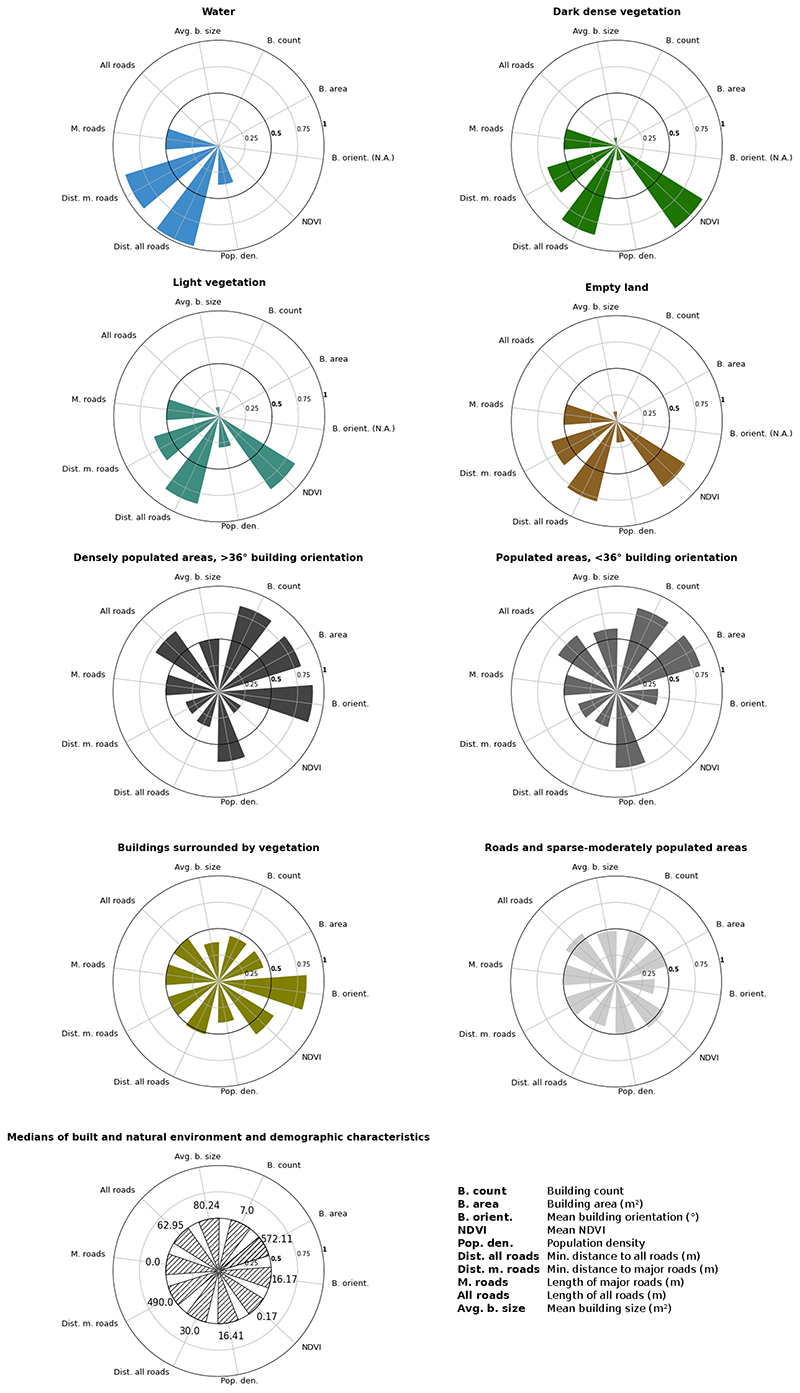
Built and natural environment and demographic characteristics of clusters. The radar charts depict the built and natural environment and demographic characteristics of each cluster. Each environmental and demographic variable is scaled with a quantile transformer, a non-parametric transformation to map the data to a uniform distribution with values between 0 and 1 (0.5 indicates the median value of a variable across all tiles in the entire image). The colours of each chart correspond to the cluster map in Fig. 2. Tiles with no buildings were included (as zeros) in summary statistics for building count and building area but excluded from calculation of summary statistics for average building size and orientation so that zero is not used in the denominator. The length of major roads was zero in 93% of all tiles, hence the median of all tiles was zero as was the mean in most clusters. NDVI: Normalised Difference Vegetation Index.

**Fig. 4:**
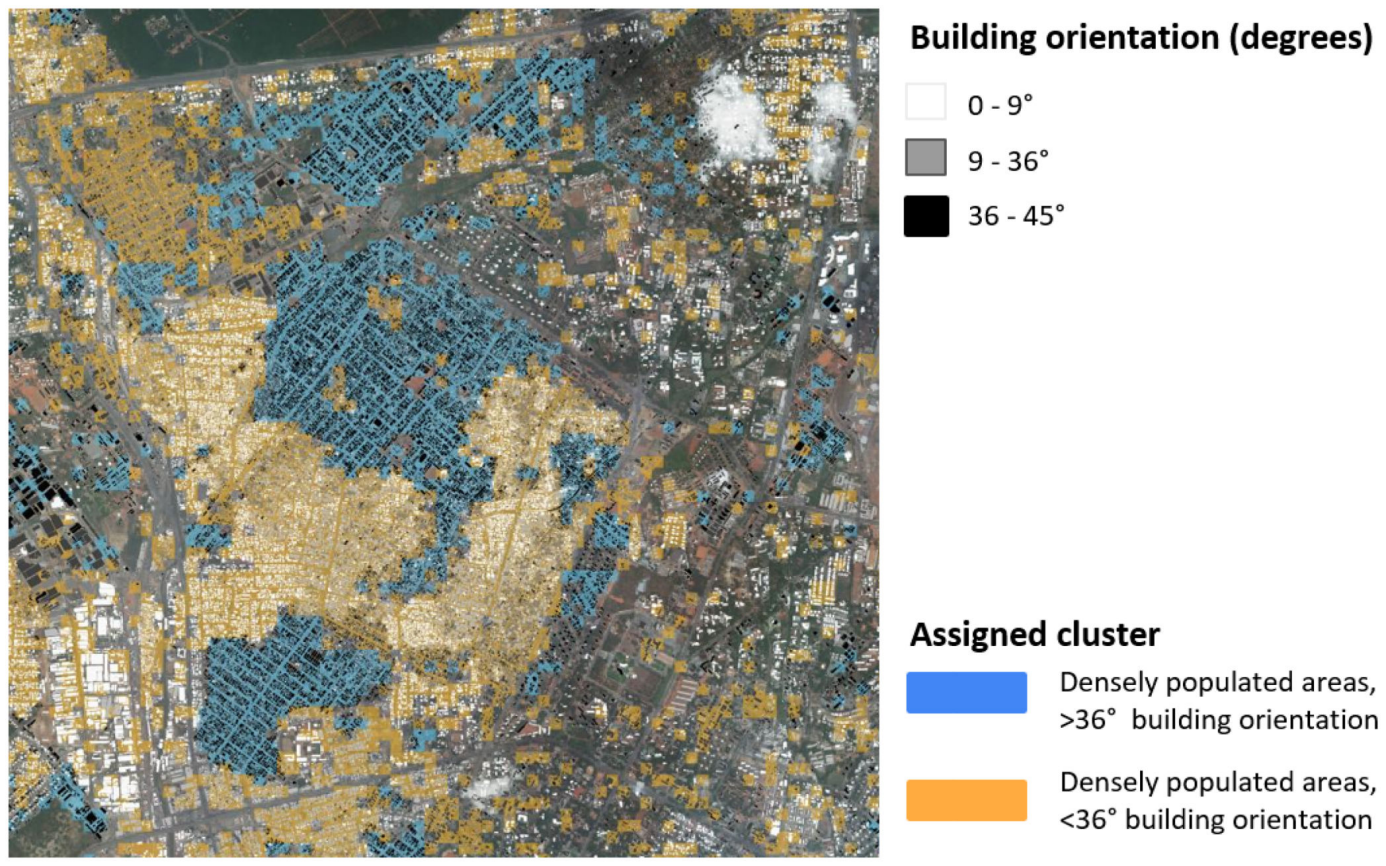
Difference in building orientation captured by the DeepCluster unsupervised analysis. The figure shows a section of the satellite image that captures the Nima and Mamobi neighbourhoods in the centre of Accra. The building shapes are plotted on top of the satellite image and are shaded by building orientation from cardinal directions. The two clusters that capture densely populated areas are shaded in orange and blue. The clusters capture a visible distinction between how the buildings are arranged within the neighbourhood.

In the main analysis, we divided GAMA into eight clusters and named each cluster as shown in Fig. 2. We further report built (buildings and roads), and natural (greenery and water) environment and demographic characteristics of the clusters which are shown in Fig. 3. Some clusters contained a single dominant characteristic with a strong visual representation. These include tiles with water, dense vegetation, and densely populated areas, especially those with distinctly visible building orientation between 36 and 45 degrees with respect to cardinal directions (as defined and calculated with data listed in Table 1 and shown in Fig. 4). The tiles that fell in these clusters had distinct distributions throughout the city, driven both by the regional environment and how the city has developed over time. The *Dark dense vegetation* cluster, which captures forest areas, is mainly located in the periphery of the city although some few patches were also present in the more urbanised Accra Metropolitan Area, for example at the University of Ghana campus. The *Water* cluster captures bodies of inland water and is often surrounded by a cluster consisting of vegetation that is less dense and lighter in colour (*Light vegetation*). The two clusters that capture densely populated areas (*Densely populated areas, >36 degree building orientation* and *Densely populated areas, <36 degree building orientation*) are mostly located in the Accra Metropolitan Area (AMA), and in adjacent metropolises, covering over one quarter of GAMA. Building-related metrics (building count, area, mean size) and population density were high in both clusters, with their distinguishing feature being building orientation with respect to cardinal directions, as defined in Table 1 and seen in Fig. 4.

The *Light vegetation* cluster and *Empty land* cluster captured natural environments that were more heterogeneous than the clusters described above. Tiles falling into the *Light vegetation* cluster varied more in their NDVI than the *Dark dense vegetation* cluster that solely captures dense vegetation in a higher NDVI range (~0.3-0.5). Tiles in the *Empty land* cluster typically had low population density and were often located next to the vegetation clusters in the northeast and northwest of the study area. Based on visual inspection, these were areas of dry soil, such as gravel, unpaved roads or sandy terrain.

Other clusters were more complex and contained multi-dimensional environmental characteristics rather than any single dominant characteristic. These include the *Buildings surrounded by vegetation* and *Roads and sparse-moderately populated areas* clusters, which together covered one half of the GAMA. The *Roads and sparse-moderately populated areas* cluster was spread throughout the city, surrounding and moving out radially from the densely populated areas in the city centre. The *Buildings surrounded by vegetation* cluster consisted of tiles that include buildings with low average building size and that were mostly located in the peri-urban areas.

### 5.2 Cluster variability in the feature space

**Fig. 5:**
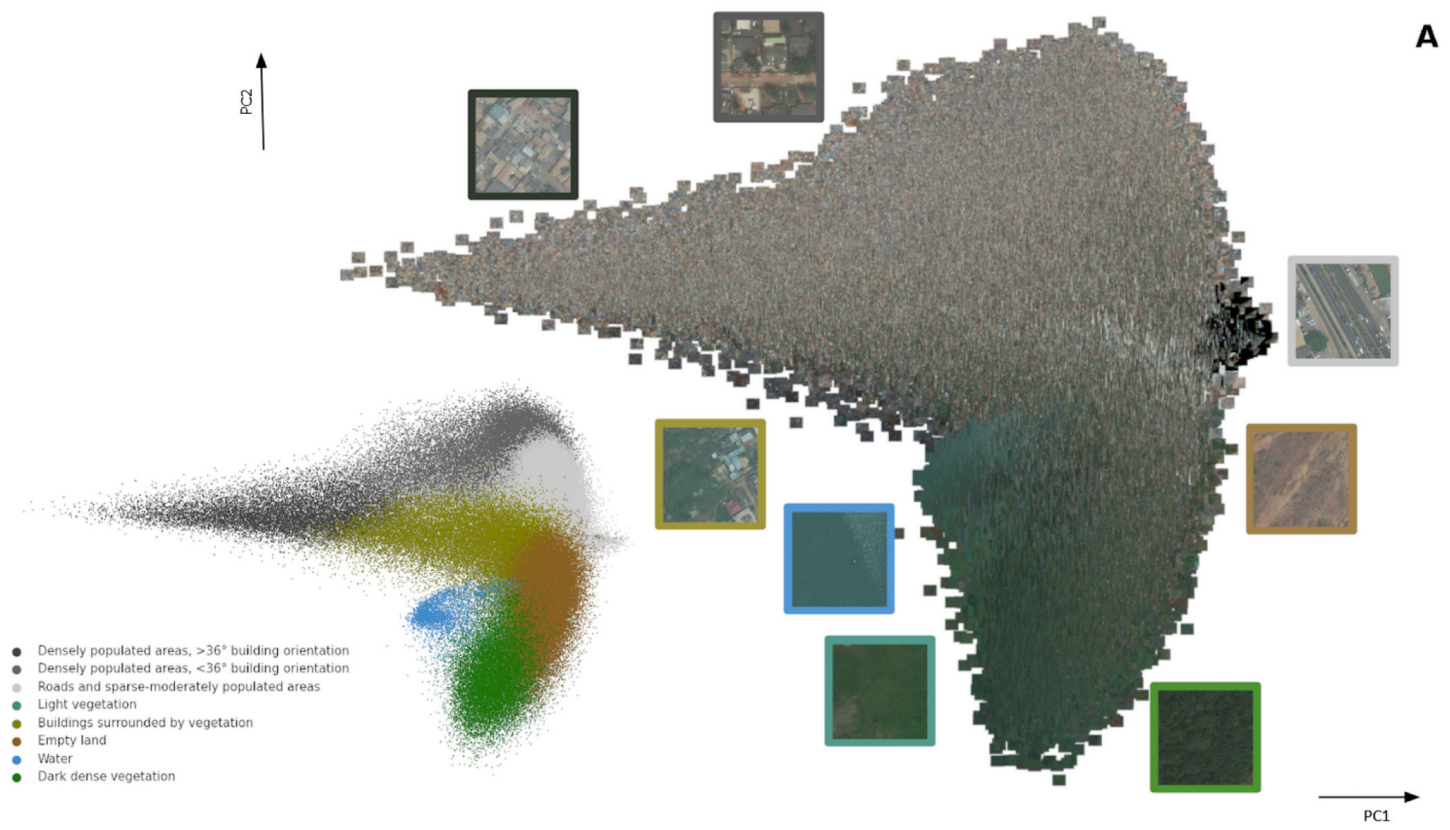

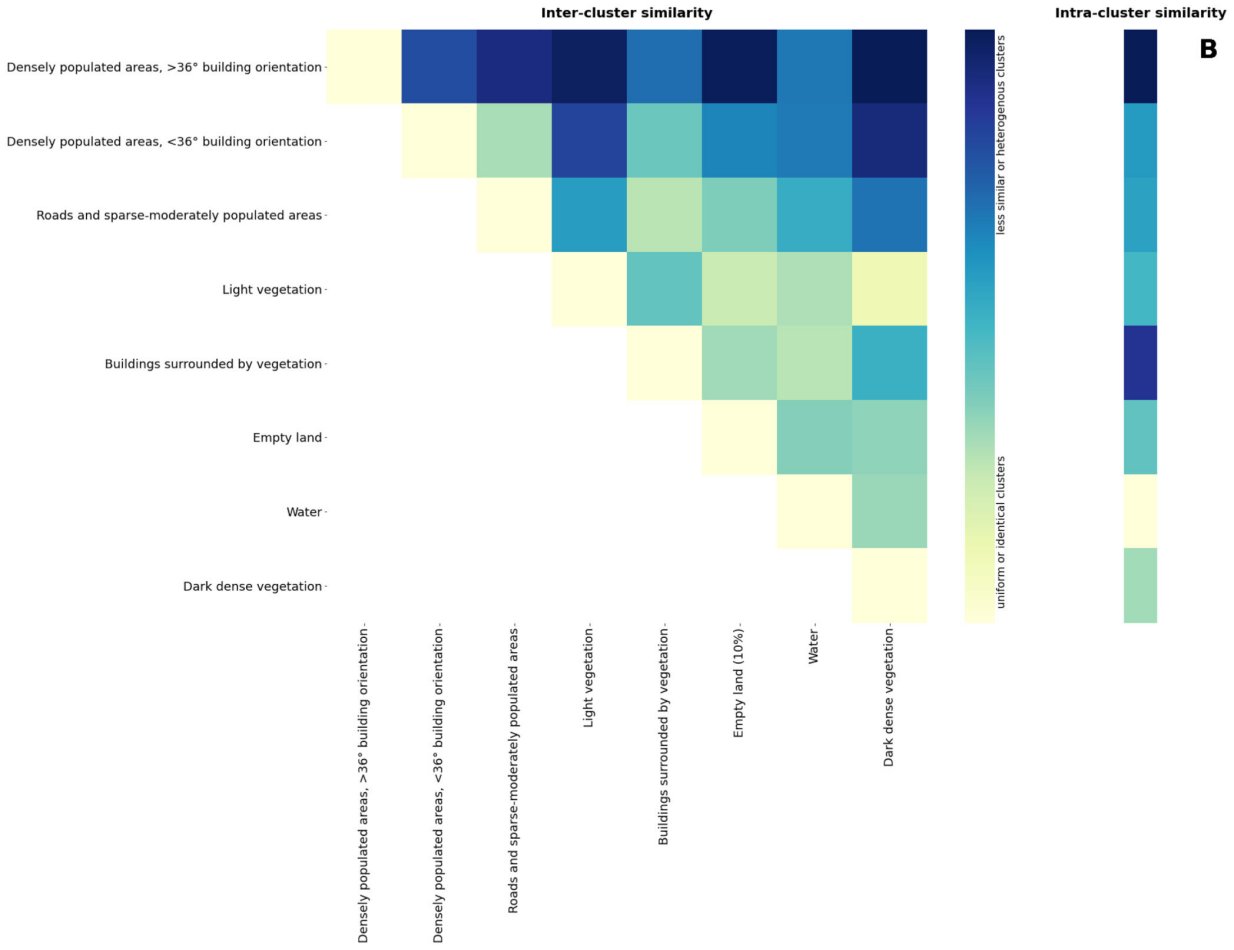
Cluster assignment and image tiles seen in the CNN-extracted feature space. Fig. 5A shows the 222,420 image tiles plotted on the first and second principal components (PCs) of 4,096 features extracted from the second to last fully connected layer of the convolutional neural network; these PCs account for 40% and 17% of the overall variance, respectively. Each tile is placed at the corresponding value of its features’ PC1 and PC2. Each tile is also shown as a point, coloured in the same colours the cluster map in Fig. 2. This presentation allows visualising image tiles, and their cluster assignment, in relation to extracted intermediate features, and can provide an intuition for how the network learns image representations in the process of cluster assignment. Fig. 5B shows average two measures of intra- and inter-cluster similarity. The intra-cluster similarity is calculated as average distance of tiles to cluster centroid, and, is a measure of within cluster uniformity (smaller distances) versus heterogeneity (larger distances). The distance between centroids of clusters is a measure of similarity across clusters. The smaller the distance between the clusters, the more similar they are, and vice versa. For computational efficiency, within- and between-cluster distances were calculated using the first two PCs.

To understand to what extent the network learns meaningful image representations used for clustering, we visualised cluster assignment and the image tiles for each cluster in the feature space (Fig. 5A) and measured the intra-cluster and inter-cluster distance (Fig. 5B), as described in Section 4.2.1. Tiles that fell into *Water* and *Dark dense vegetation* clusters had small intra-cluster distance which indicates they were very homogeneous, and those in *Densely populated areas, >36 degree building orientation* and *Buildings surrounded by vegetation* cluster were highly variable (Fig. 5B); other clusters fell between the two groups in terms of their intra-cluster similarity versus variability. We also measured the inter-cluster similarity by calculating the average distance between each pair of cluster centroids in the feature space. The clusters that captured the natural environment (*Water*, *Dark dense vegetation, Light vegetation*, and *Empty Land)* were close to one another in the feature space. The *Densely populated areas, >36 degree building orientation* and *Densely populated areas, <36 degree building orientation* clusters were furthest apart from the water and vegetation clusters, and the *Buildings surrounded by vegetation* and *Roads and sparse-moderately populated areas* clusters were intermediate distance to these groups. The clusters that contained multi-dimensional environmental characteristics, *Buildings surrounded by vegetation* and *Roads and sparse-moderately populated areas*, were also close to each other in the feature space.

### 5.3 Prediction of cluster assignment with external variables

**Fig. 6:**
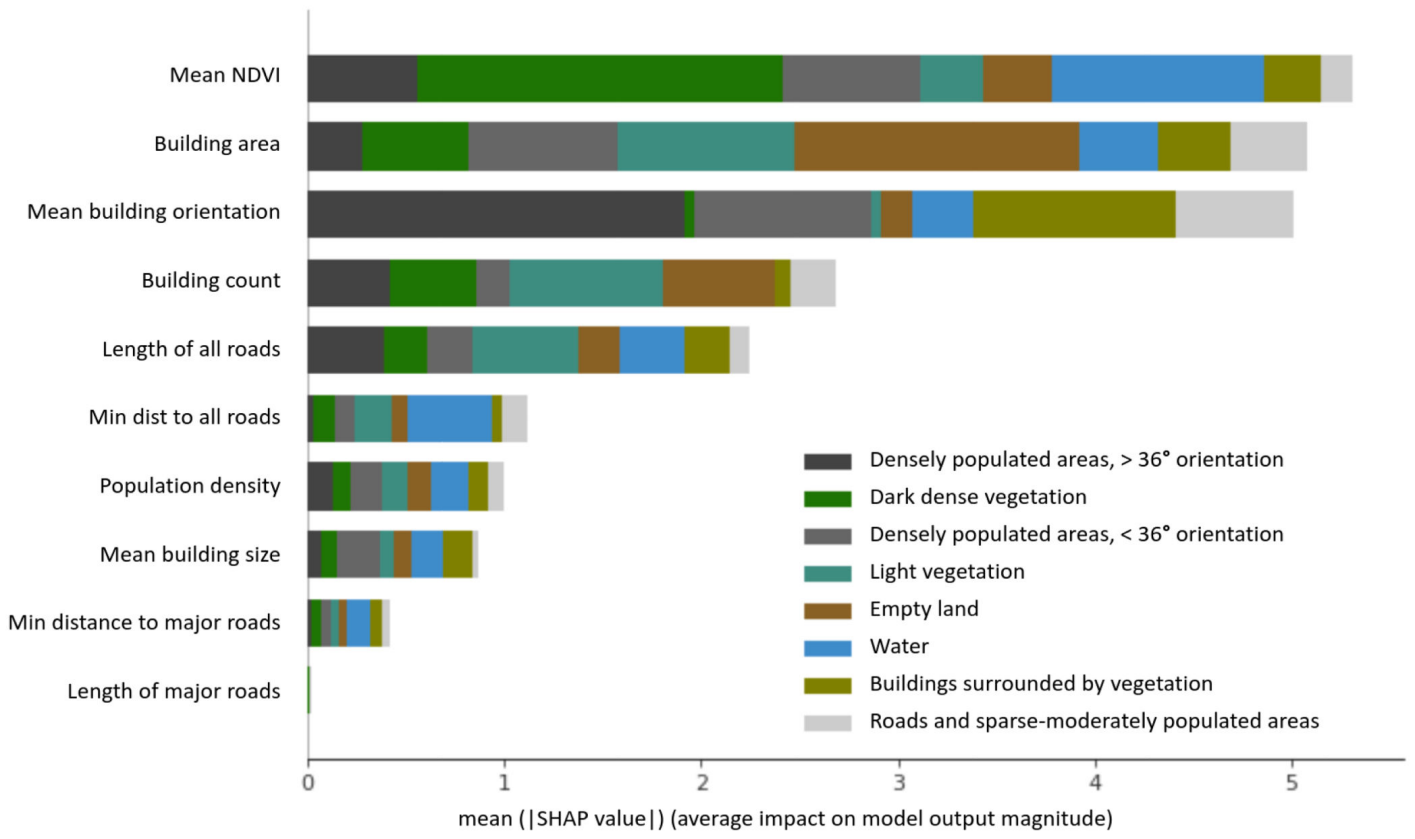
Built and natural environment and demographic variables as predictors of membership in image-based clusters. The figure shows SHapley Additive exPlanations (SHAP)^59^, obtained by fitting XGBoost^57^ classifier to predict cluster membership by environmental and demographic variables. The SHAP value for each variable indicates its predictive power for assignment to various clusters, and hence identify the measures of urban form (buildings and roads), environment (water and vegetation), and population that differentiate clusters that were generated based on images alone. The mean SHAP values from the XGBoost classifier were calculated for each environmental and demographic variable as described in Methods. The total length of each bar, which is the mean absolute SHAP value, represents the overall importance of each variable for predicting cluster membership, and the different colours represent the importance for assignment to each cluster.

To quantitatively evaluate what features of urban form, environment and population are most represented in the visually identified clusters, we trained a machine learning classifier to predict cluster membership using environmental and demographic variables not used in clustering, as described in Section 4.3. Fig. 6 shows the SHAP values, which are a measure of variable importance for describing cluster assignment. A higher SHAP score indicates larger relevance of a certain variable on predicting cluster assignment. The SHAP values show that NDVI was an important external predictor for image-driven cluster membership, especially through its substantial role for identifying the *Water* (which had very low NDVI) and *Dark dense vegetation* (very high NDVI) clusters. At the same time, other variables helped predict cluster assignment beyond the role played by NDVI. Specifically, NDVI was followed by mean building area, whose most salient role was in predicting *Empty land* and the two vegetation clusters (which all had very low building area), and building orientation, whose importance was driven most by its ability to predict *Densely populated areas, >36 degree building orientation*. Tiles belonging to the heterogeneous clusters of *Roads and sparse-moderately populated areas* and *Buildings surrounded by vegetation* were not predicted by any single environmental variable but rather had contributions from multiple ones. Distance and length of major roads were the least relevant variables in predicting cluster memberships, likely because their role was already captured in the same metrics for all roads. Population density and mean building size were ranked comparatively low as well, possibly because the information on building size was already captured by building area and count, and population density was moderately correlated with building-related metrics (Appendix Fig. A.2). This happens because the SHAP importance score is partitioned such that correlated features will not rank as highly as when their impacts were considered in isolation.

### 5.4 Sensitivity analyses

#### 5.4.1 Sensitivity to image tile dimensions

We investigated cluster stability with varying tile size, specifically 128 x 128 (i.e. 50% smaller than main analysis in side length), 384 x 384 (50% larger), and 512 x 512 (100% larger) pixels, as described in Section 4.4.1. Analyses with different tile sizes showed that tiles with single, dominant characteristics such as vegetation, water, and densely populated areas, were clustered largely independently of tile size (Appendix Fig. A.3). The resultant clusters were stable in their (external) characteristics and in terms of areas they cover, supported by the co-occurrence plots that compare cluster membership between the different tile sizes (Appendix Fig. A.4). Tiles with multi-dimensional environmental characteristics in the main analysis, such as the *Buildings Surrounded by Vegetation* and *Roads and sparse-moderately populated areas* clusters, varied the most depending on the tile size and areas of the image they are assigned to. Smaller tiles (128 x 128 pixels) captured more homogenous landscapes and less mixed environments than the original and larger tile sizes, such as *Buildings surrounded by vegetation*. In contrast, larger tiles (384 x 384 pixels, 512 x 512 pixels) were more likely to capture mixed environments, such as sparse-moderately populated areas and buildings and roads together with vegetation. These two clusters make up 49% and 54% of the total tiles for the analysis with 384 x 384 pixels and 512 x 512 pixels, respectively, compared to 39% in the 128 x 128 pixel analysis.

#### 5.4.2 Sensitivity to the number of clusters

**Fig. 7:**
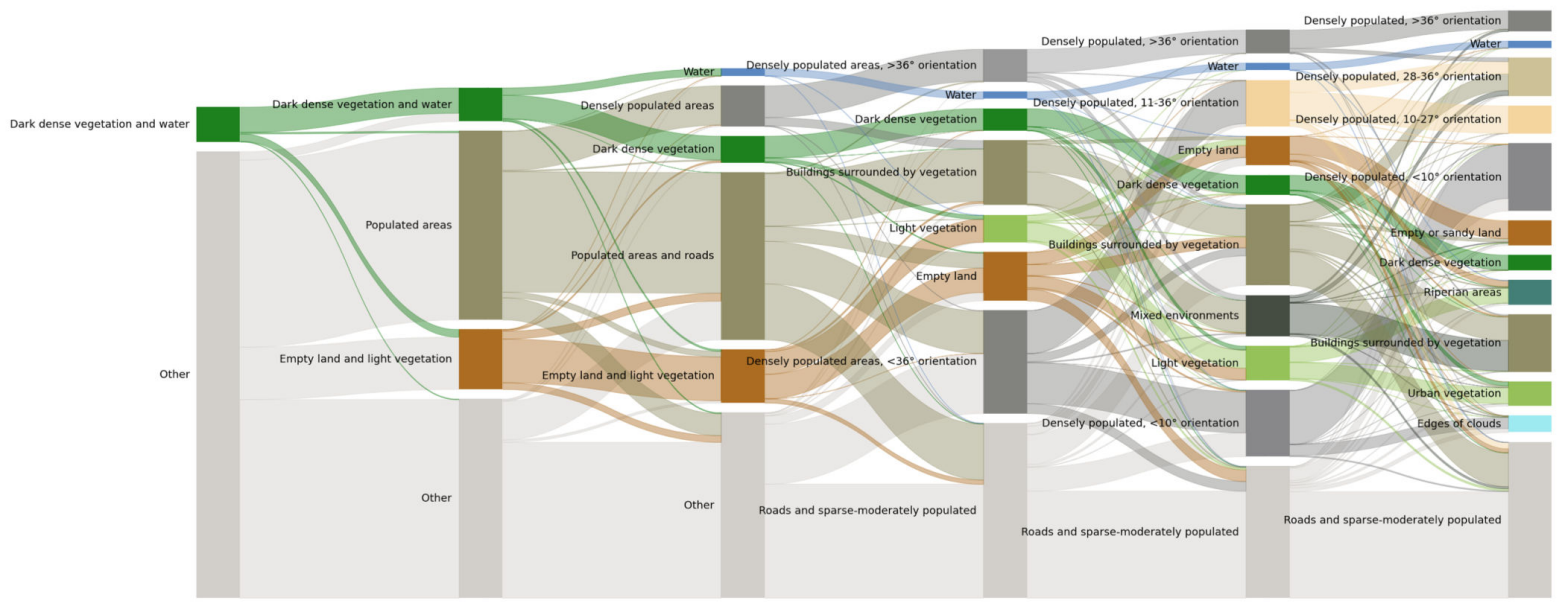
Impact of cluster number on the clustering outcome. The Sankey plot shows the sizes of clusters when cluster number (K) varied from 2 to 12. The flow of data indicates how clusters partition and merge based on repeating the main analysis with varying K. The naming of the clusters for each K is based on a similar process as in the main analysis, and based on the environmental and demographic characteristics of each cluster.

As part of the sensitivity analysis, described in Section 4.4.1, we analysed how the clusters changed when we set the cluster number, K, from K=2 to K=12 (Fig. 7). The Sankey plot shows that initially (K=2), the tiles were separated into a cluster containing two very homogenous natural environments (water and dark dense vegetation) and a cluster that captured all the other tiles. At K=4, that mixed cluster split into clusters with distinct visual character including populated areas and empty land and light vegetation. The natural environment cluster that formed at K=2 further split into dark green vegetation and water tiles at K=6. At K=6, the algorithm also grouped densely populated areas with high building orientation together, a cluster that stays constant until K=12. Clusters capturing mixed environments such as roads and sparse-moderately populated areas and buildings surrounded by vegetation appeared later at K=8. By K=10 and K=12, very particular clusters appear, such as a cluster that captures populated areas with very low building orientation (<10 degrees), a cluster capturing riparian areas, and a cluster that contained edges of clouds. The results show that clusters that consisted of a single defining characteristic (e.g., dense vegetation or densely populated areas) were more robust to the choice of cluster number, whereas these based on a combination of characteristics (e.g., buildings surrounded by vegetation) changed more based on the number of clusters. Comparing the cluster development with the cluster homogeneity in the extracted feature space (Fig. 5), the clusters that emerge first (dark and light vegetation and water) were also the clusters which were most internally uniform as shown by the feature plot (Fig. 5B).

#### 5.4.3 Influence of hyperparameter k on feature learning

To investigate how the hyperparameter k influences deep feature creation, we compared the cluster results of three sets of deep features (DF) that were learnt with different choices of hyperparameter k (k ∈ {8, 50, 100}), namely DF_k8_, DF_k50_, and DF_k100_, as described in Section 4.4.2. The visual interpretation through the radar plots of the three sets of eight clustered deep features (Appendix Fig. A.5) and the co-occurrence of cluster assignments (Appendix Fig. A.6) showed that the tiles were mostly grouped in similar ways for different values of k and had consistent environmental and demographic characteristics. Each choice of k included a *Water, Dark dense vegetation, Light Vegetation*, and *Densely populated areas with >36 degree building orientation and <36 degree building orientation* cluster. The main changes between cluster results were in the *Empty land* cluster and clusters with the mixed environments, the *Buildings surrounded by vegetation* and the *Roads and sparse-moderately populated areas* clusters, which differed in building area and count, as well as building orientation. The building area and count of the clusters formed from the DF_k50_ and DF_k100_ were slightly higher than the cluster results from DF_k8_ for the *Roads and sparse-moderately populated areas* cluster, but slightly lower for the *Buildings surrounded by vegetation* cluster. This change arose mainly due to the fact that more tiles were assigned to the *Roads and sparse-moderately populated areas* and *Buildings surrounded by vegetation* clusters than in the main analysis, with 60% of tiles being assigned to these two clusters in the DF_k50_ analysis and 69% in the DF_k100_ analysis, compared to 50% in the main (DF_k8_) analysis. The additional tiles for the *Buildings surrounded by vegetation* mainly came from the *Empty land* cluster in the main analysis (Appendix Fig. A.6). Furthermore, the remaining part of the *Empty land* cluster created with DF_k50_ and DF_k100_ also contained some vegetation compared to the clusters formed in the main (DF_k8_) analysis.

## 6 Discussion

Cities are complex dynamic systems whose built and natural environments, including buildings, roads and vegetation, are shaped through an interplay of local geography and human activity. These environments in turn affect where people live and conduct their activities, how they commute among these places, and their impacts on health and wellbeing. Our analysis showed that application of unsupervised clustering can capture single- and multi-feature urban environments and hence offer a novel way of coherently and comprehensively characterising and tracking urban environmental change, especially in settings where labelled data are limited.

### 6.1 Implications for tracking sustainable urban development

Our results show that the image-based clusters present interpretable insights into neighbourhoods. The proposed framework can be used to track changes in the built and natural environment at a fine spatial scale and in near-real-time to inform urban planning and services. The clusters capturing vegetation and water, which have distinct visual features, are influenced by regional geography and whether and how it is preserved or modified. The *Dark dense vegetation* cluster captures the hills and valleys in the north of the GAMA, where human activities have so far been relatively minimal. These forests used to cover even larger parts of north and northwest regions of the GAMA, but land clearing for urban growth and agriculture, charcoal making and firewood collection have substantially reduced the extent of trees^49,63^. The *Water* cluster covers waterways and other water bodies, and is surrounded by *Light vegetation* cluster, which lies closer to human settlements than the *Dark dense vegetation* cluster. In addition to riverine vegetation, the *Light vegetation* cluster captures a wetland that separates the Accra Metropolitan Area from the adjacent metropolis of Tema in the east, as well as protected areas around a reservoir. These areas face threats of urban encroachment, and their conservation is necessary for preservation of the region’s biodiversity and to protect the city from flooding^10,64–67^. In particular, large parts of Accra’s informal settlements (which fall in our two densely populated clusters) are located next to riparian areas and are exposed to a risk of flooding^68,69^, which is predicted to increase due to global climate change^65^. The *Empty land* cluster captures open unvegetated land, such as sandy or bare soil with a few shrubs or unfinished buildings and unpaved paths. Empty land that has recently lost vegetation cover may be a setting for imminent road and building construction and could indicate the beginning of city sprawl^70^. These changes, and how they change the city’s environment, can be readily monitored with sequential satellite images and our clustering approach.

The two densely populated areas (Densely populated, >36 and <36 degree building orientation) are mostly located in the dense core of the city and adjacent metropolis, a feature that is seen in many cities in Africa^71^. These densely populated areas tend to be poorer than other parts of the city but are well-connected to urban transport and trade hubs, which makes them a setting for informal and formal business and trade activities. The dense population and the commercial activities create more social cohesion but also make these areas noisier and more polluted^72–74^. Despite currently having no vertical layering, both clusters are dense in terms of building footprint, as is the case in informal settlements in other cities in the developing world^75,76^. There is a trend by private and public-private developers towards larger and higher buildings in these highly accessible locations, which contrasts with earlier urban sprawl in Accra and other major African cities^18,77^. Vertical densification can increase economic productivity, while also displacing their current residents unless accompanied with appropriate housing in the same or nearby locations as a part of redevelopment. It will also likely change the visual characteristics of these areas and hence can be measured and monitored through the approach that we presented. The main difference between the two densely populated clusters is the visually distinct building orientation (Fig. 4), which impacts thermal comfort. This feature which will be increasingly relevant as extreme weather events, especially high temperatures, become more frequent with changing climate^78–81^.

The remaining clusters capture a more complex and heterogeneous mix of land cover and land use. The areas captured by the *Buildings surrounded by vegetation* cluster are either farmland at the fringe of the metropolitan area or wealthier neighbourhoods with freestanding houses surrounded by gardens, often as part of gated communities. These areas have lower pollution^72–74^ and benefit from proximity to greenspace. The parts of the cluster that are at the fringe of the city are expected to grow further into the surrounding natural environments^82^, driven by population growth, cheaper housing stock compared to the city centre, and poorly controlled private land development due to weak enforcement of urban planning and development rules^10,83^. Limiting this sprawl requires a combination of land tenure reforms, and introduction/enforcement of urban planning and zoning regulations, so that outward growth and sprawl are balanced with (vertical) densification of already-built areas as described earlier^10,84^. Finally, the *Roads and sparse-moderately populated areas* cluster represents the combination of the city’s low-medium density residential and commercial settlements and its road network. Road capacity in GAMA is inadequate for the increasing number of vehicles, and the peri-urban areas are underserved^85^, as evidenced by the relative underrepresentation of *Roads and sparse-moderately populated areas* cluster in the peri-urban areas, especially in the northeast of GAMA. The combination of this cluster, the two densely populated clusters and the *Buildings surrounded by vegetation* cluster, captures most of the paved and unpaved roads, and provides a good representation of the connectivity and accessibility of the city. The arrangement of these clusters can help to identify areas that are poorly connected, and reveal options for improving their connectivity including walkable and bikeable areas, those that can be connected to central Accra with radial rapid rail or bus transport systems, and those that may require additional roads^56,87^.

### 6.2 Application and extension to other cities and multiple time points

The unsupervised approach can be applied to other cities to reveal similarities and differences in the character of natural and built environments. Similarly, the framework can be used for longitudinal analysis of satellite images taken at different points in time in order to track urban change based on how each phenotype expands or replaces others.

The main consideration for application to another single city is the number of clusters, which should be adapted to to the local environmental context as well as application. A lower number of clusters (K) can aid in distinguishing the built and natural environment, and higher number of clusters can highlight more specialised phenotypes that capture mixed environments. Further methodological considerations are needed for extensions to multiple cities and time points. First, researchers must consider whether to cluster cities together or separately. Separate clustering will allow place-specific clusters to arise. However, clusters of different cities are not directly comparable. In contrast, joint clustering will create comparable clusters but may not pick a feature of built or natural environment that is unique to a specific city. Similarly, the choice of number of clusters needs to balance the comprehensiveness of clusters and their interpretability, especially when cities are clustered jointly.

### 6.3 Strength and limitations

We presented a novel approach for using high-resolution satellite imagery without external data to capture variations in multiple natural and built environment of cities and can provide timely data to support sustainable and healthy urban development. The approach coherently integrates different features of the urban environment, which have traditionally been analysed in isolation. The algorithm combines feature extraction and clustering in one model, making its implementation easy and efficient. We used very high-resolution satellite imagery, which shows objects as small as cars and trees, and therefore allows the model to use information on high level features of the urban environment. The results were interpreted against built and natural environment and demographic characteristics that demonstrate the interpretability of the model to help its wider use. We also investigated the image representations in the feature space to better understand the cluster formation and assessed the sensitivity of the approach to key choices such as tile size and cluster number. The approach used here picks up more detailed clusters than pixel-level analysis (e.g., land classification by NDVI) could do. For example, the two densely populated areas clusters had very similar median NDVI, however, but differed in building orientation and average building size. Similarly, the *Buildings surrounded by vegetation* and the *Empty land* clusters had similar median NDVI but visually look very different. The former contains a mix of built environments and natural environments, whereas the latter did not have any built structures and had little or no vegetation. The SHAP analysis (Fig. 6) supported this observation and showed that factors beyond NDVI help with separation of image-based clusters.

The main limitations of the analysis are related to the geographic and temporal data availability. A direction of further research should therefore either analyse additional points in time or additional cities. We used one satellite image that captured the city at a specific point in time, although weather and season could have an impact on pixel intensities. While the data on built and natural environment and demographic characteristics were obtained in the same year, there could be a mismatch between the exact date the satellite image was taken and these data were gathered or estimated. Additionally, the analysis was limited by the datasets used to interpret the clusters. Further data on the built and natural environment, such as on building height or agricultural land, might have improved the cluster interpretation. Finally, the CNN-based tile-level approach is computationally more costly than a simpler pixel-level analysis. However, as stated above, our framework is able to capture more detailed information beyond what is captured by one pixel. As computing power increases and becomes available at low cost, analysis will be become faster and more accessible.

## 7 Conclusions

Cities create opportunities to reduce poverty, improve health and wellbeing, and enhance local and global sustainability^3,4,88,89^. To develop and refine policies that leverage the potential of expanding cities in Africa for sustainable development, it is essential to track the extent and characteristics of urban growth and change at different scales, which is currently hindered by the quantity and fragmented nature of available data. Our work shows that unlabelled satellite images together with unsupervised deep learning have the potential to bridge the data gaps that exist on temporal and spatial scales and provide a scalable approach for tracking urban development throughout the developing world. This approach will become increasingly cost-effective and efficient as satellite images become more accessible and affordable, and computing power increases. It can be automated to track cluster changes between different time points at near real-time speed in different cities. In doing so, our approach can help bridge the data gap between the developing and industrialised nations, and provide a more equitable deep learning approach that does not rely on labelled image data that are largely gathered in the industrialised world^90^.

## Supplementary Material

Appendix

## Figures and Tables

**Table 1:** Sources of data on built and natural environment and population in Accra.

Environmental or demographic variable	Unit	Data type	Year	Source
Building area	m^2^	Vector	2019	Maxar and Ecopia.ai^1^
Building count	integer	Vector	2019	Maxar and Ecopia.ai^1^
Average building size	m2	Vector	2019	Maxar and Ecopia.ai^1^
Average building orientation	degrees (°)	Vector	2019	Maxar and Ecopia.ai^1^
Length of major roads	m	Vector	2019	OpenStreetMap^2^
Length of all roads	m	Vector	2019	OpenStreetMap^2^
Minimum distance to major roads	m	Vector	2019	OpenStreetMap^2^
Minimum distance to all roads	m	Vector	2019	OpenStreetMap^2^
Population density	estimated total number of people per grid-cell	Raster (~100 m/pixel)	2019	WorldPop^3^
Mean NDVI	index (-1 to +1)	Raster (~30 m/pixel)	01-01-2020	Landsat^4^

1 https://ui.adsabs.harvard.edu/abs/2019AGUFMIN11D0688H/abstract. The building information is provided in a vector format. We overlaid the vector with a grid that represents the tile size and location. For each measure, we calculated the mean value per tile. Building orientation was computed with the momepy package^91^ as deviation of orientation from cardinal directions; it was defined as an orientation of the longest axis of the bounding rectangle in range 0-45 degrees. Building orientation is measured with respect to cardinal directions, and is a visual feature of the built environment. An example is shown in Fig. 4, in which we plotted a section of the satellite image together with the building shapes (coloured by building orientation) and assignment to two clusters that are similar in most characteristics, except building orientation. Building orientation also has a physical relevance for residents, impacting the natural lighting and ventilation.

2 https://www.openstreetmap.org/. The road information is provided in a vector format. We overlaid the vector with a grid that represents the tile size and location, and calculated statistics per tile.

3 https://www.worldpop.org/geodata/summary?id=6116. We used a population raster with a resolution of 100 m to calculate the mean population density per tile. It was computed by vectorising the population density raster file, overlaying it with the tile grid and calculating the mean per tile.

4 https://www.usgs.gov/centers/eros/science/usgs-eros-archive-landsat-archives-landsat-8-oli-operational-land-imager-and?qt-science_center_objects=0#qt-science_center_objects. We use Landsat imagery from 01/01/2020, a cloudless day, to calculate the mean NDVI value for each tile.

## Data Availability

All data used for the analysis are openly available and data sources are listed in the data table (Table 1). Code will be made available on the *Pathways to Equitable Healthy Cities* research collaboration website (https://equitablehealthycities.org/data-download/) upon publication of the paper. The DeepCluster algorithm, which was published by Facebook research and is also openly available, was run on 3 RTX6000 GPUs, 72GB memory and a runtime of approximately 24 hours.
