# Supplementary material for: Phenotyping urban built and natural environments with high-resolution satellite images and unsupervised deep learning
Source: Sci Total Environ. Author manuscript; Available in PMC 2023 Oct 1. (PMC7615085; doi:10.1016/j.scitotenv.2023.164794)
Supplement: Appendix [file EMS187523-supplement-Appendix.pdf]

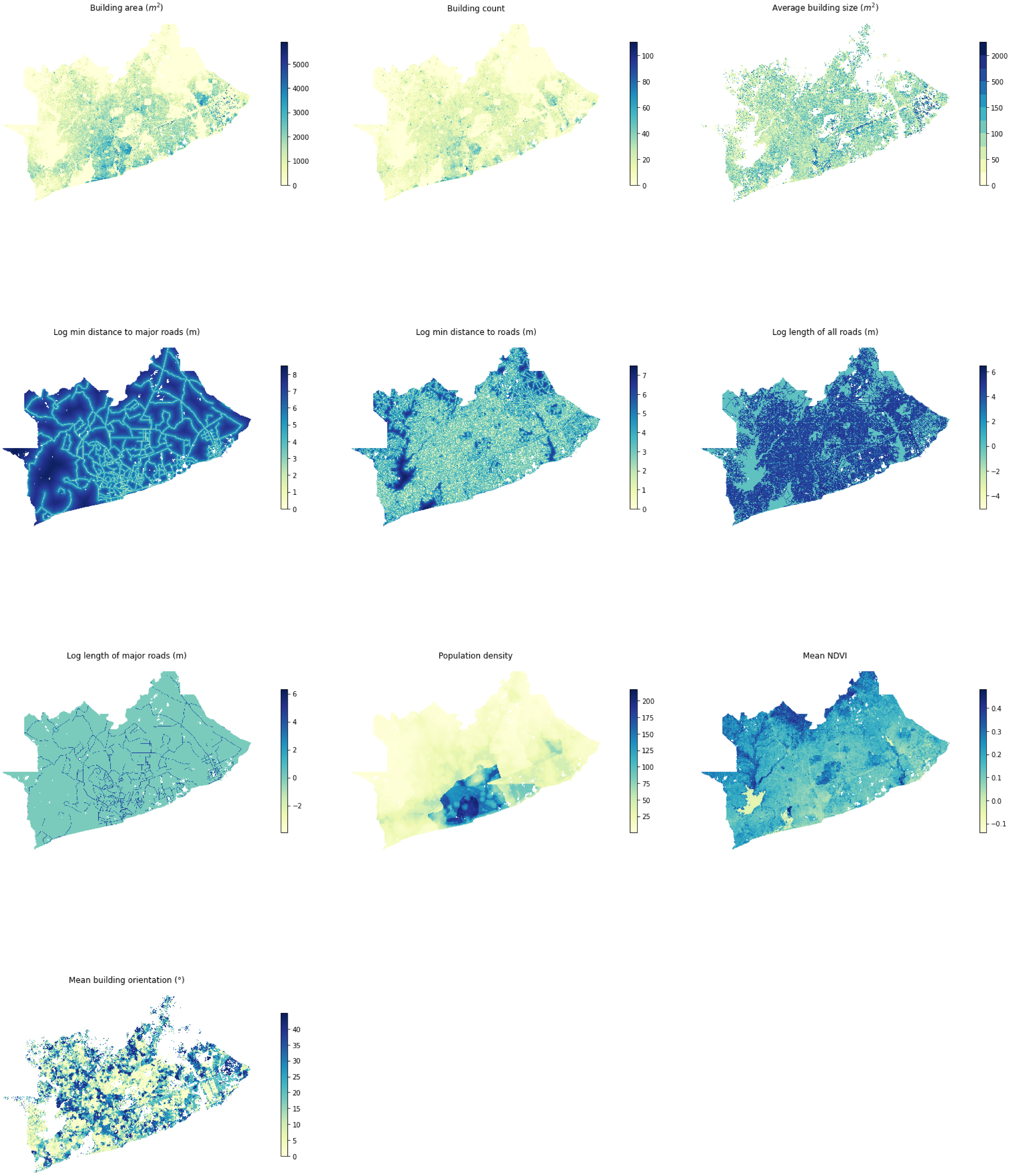

Building area (m^2)
0 1000 2000 3000 4000 5000
Building count
0 20 40 60 80 100
Average building size (m^2)
0 50 100 150 500 2000
Log min distance to major roads (m)
0 1 2 3 4 5 6 7 8
Log min distance to roads (m)
0 1 2 3 4 5 6 7
Log length of all roads (m)
−4 −2 0 2 4 6
Log length of major roads (m)
−2 0 2 4 6
Population density
25 50 75 100 125 150 175 200
Mean NDVI
−0.1 0.0 0.1 0.2 0.3 0.4
Mean building orientation (°)
0 5 10 15 20 25 30 35 40

Appendix

Appendix Fig. A.1: Maps of built and natural environment and demographic variables in the Greater Accra Metropolitan Area.

The map shows each variable for the same tiles as used for clustering.

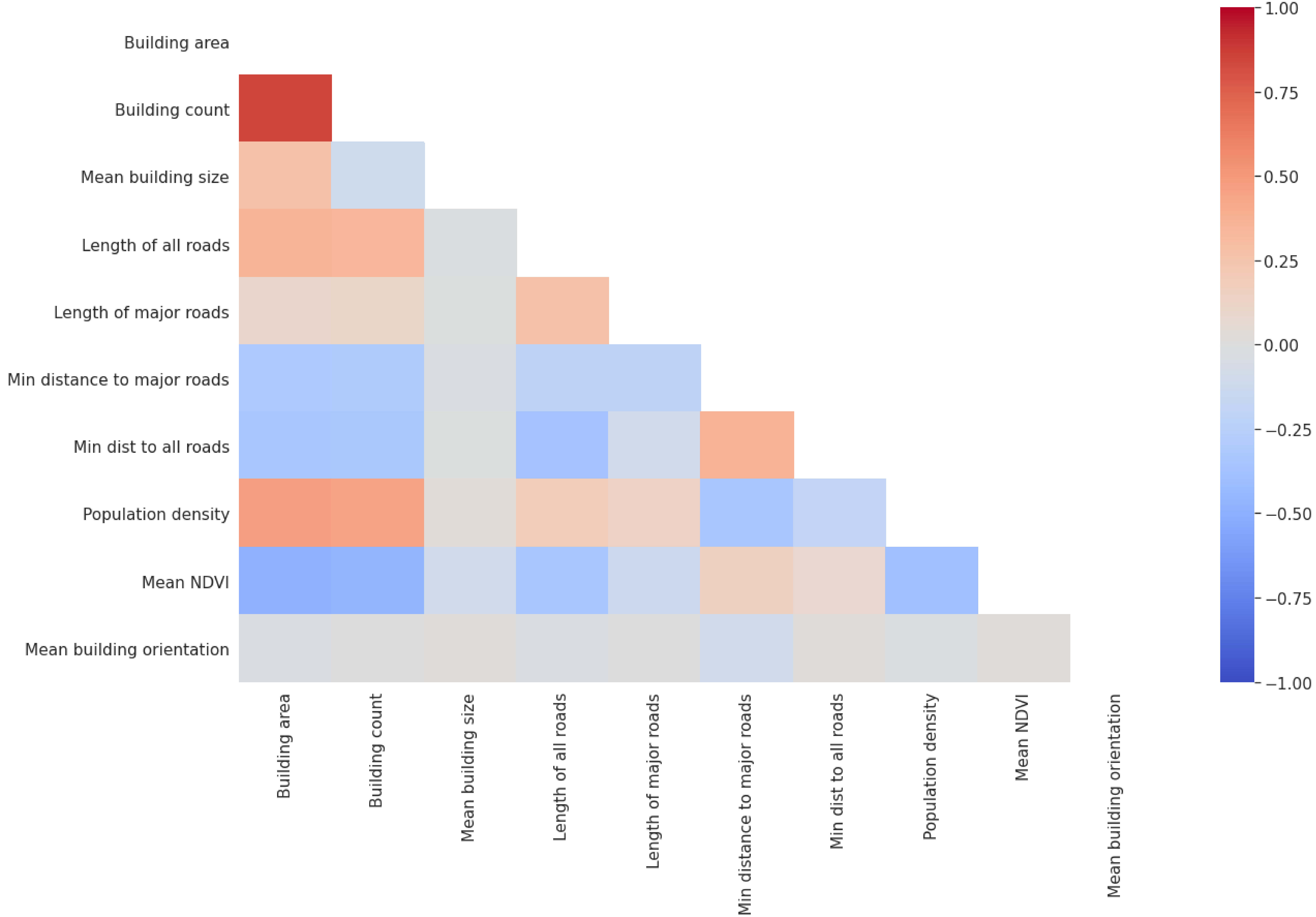

Building area
Building count
Mean building size
Length of all roads
Length of major roads
Min distance to major roads
Min dist to all roads
Population density
Mean NDVI
Mean building orientation
1.00
0.75
0.50
0.25
0.00
−0.25
−0.50
−0.75
−1.00

Appendix Fig. A.2: Correlation of environmental and demographic variables used to interpret the clusters.

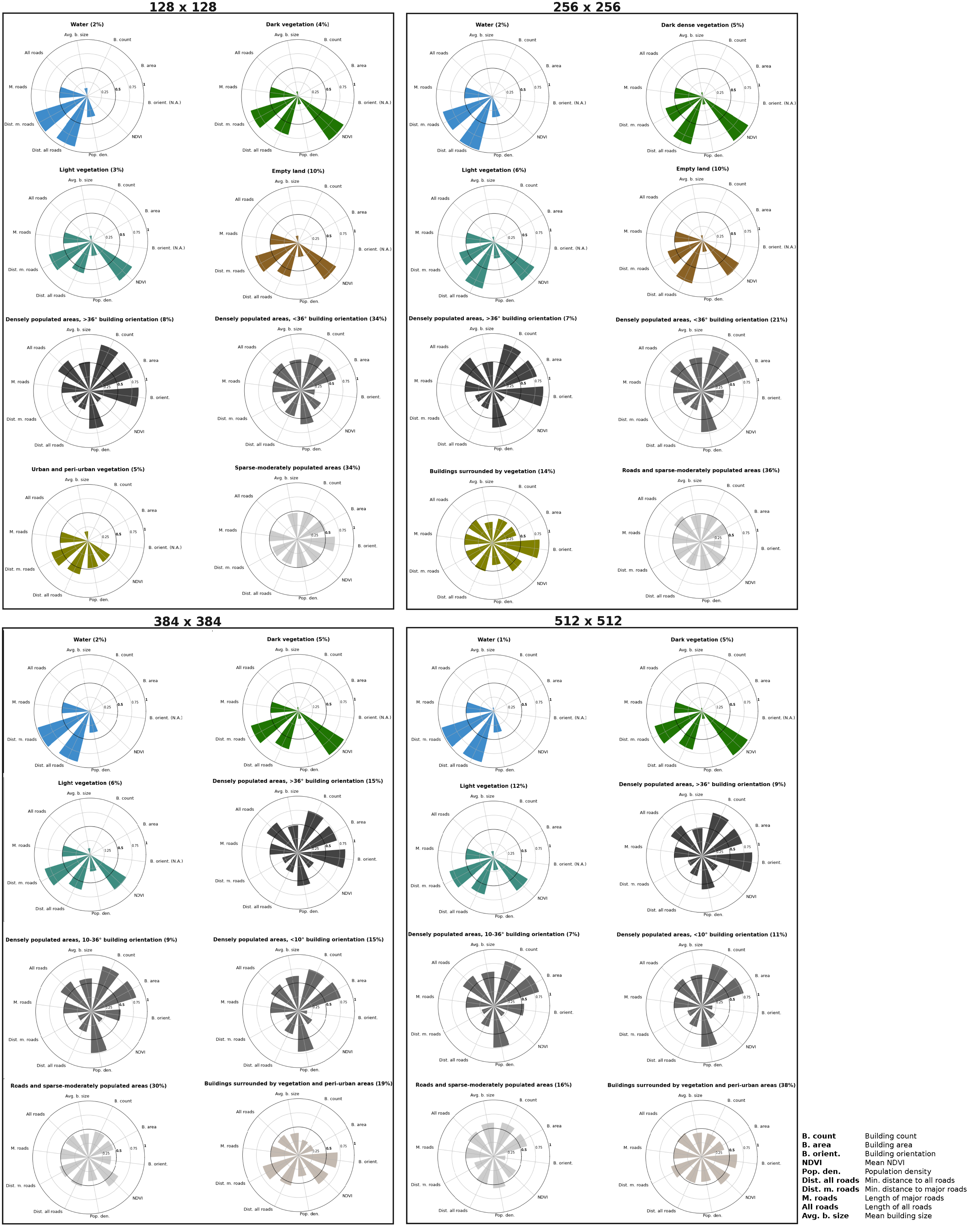
128 x 128
Water (2%)
Dark vegetation (4%)
Light vegetation (3%)
Empty land (10%)
Densely populated areas, >36° building orientation (8%)
Densely populated areas, <36° building orientation (34%)
Urban and peri-urban vegetation (5%)
Sparse-moderately populated areas (34%)
256 x 256
Water (2%)
Dark dense vegetation (5%)
Light vegetation (6%)
Empty land (10%)
Densely populated areas, >36° building orientation (7%)
Densely populated areas, <36° building orientation (21%)
Buildings surrounded by vegetation (14%)
Roads and sparse-moderately populated areas (36%)
384 x 384
Water (2%)
Dark vegetation (5%)
Light vegetation (6%)
Densely populated areas, >36° building orientation (15%)
Densely populated areas, 10-36° building orientation (9%)
Densely populated areas, <10° building orientation (15%)
Roads and sparse-moderately populated areas (30%)
Buildings surrounded by vegetation and peri-urban areas (19%)
512 x 512
Water (1%)
Dark vegetation (5%)
Light vegetation (12%)
Densely populated areas, >36° building orientation (9%)
Densely populated areas, 10-36° building orientation (7%)
Densely populated areas, <10° building orientation (11%)
Roads and sparse-moderately populated areas (16%)
Buildings surrounded by vegetation and peri-urban areas (38%)
Avg. b. size
B. count
B. area
B. orient. (N.A.)
B. orient.
NDVI
Pop. den.
Dist. all roads
Dist. m. roads
M. roads
All roads
0.25
0.5
0.75
1
B. count Building count
B. area Building area
B. orient. Building orientation
NDVI Mean NDVI
Pop. den. Population density
Dist. all roads Min. distance to all roads
Dist. m. roads Min. distance to major roads
M. roads Length of major roads
All roads Length of all roads
Avg. b. size Mean building size

Appendix Fig. A.3: Impact of image tile size on urban cluster characteristics.

The radar charts show the built and natural environment and demographic characteristics of each cluster per analysis with tile sizes: 128 x 128 pixels, 256 x 256 pixels (main analysis), 384 x 384 pixels, and 512 x 512 pixels. Each environmental and demographic variable is scaled with a quantile transformer, a non-parametric transformation to map the data to a uniform distribution with values between 0 and 1 (0.5 indicating the median value of that certain feature across all tiles in the entire image). Tiles with no buildings were included in summary statistics for building count and building area (as zeros) but excluded from calculation of summary statistics for average building size and orientation so that zero is not used in the denominator.

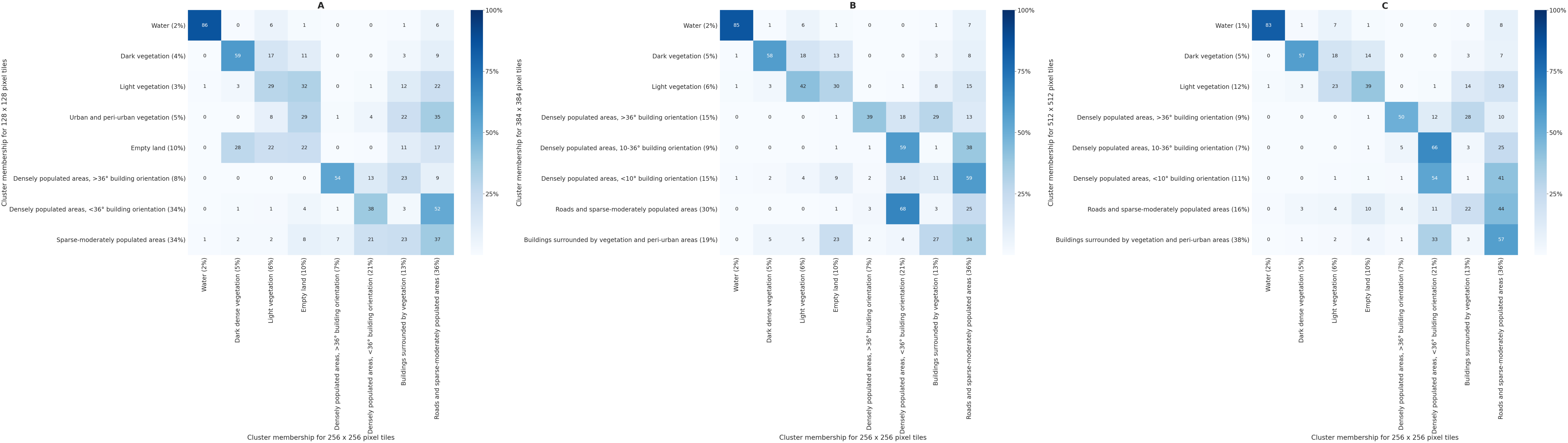

A
Cluster membership for 128 x 128 pixel tiles
Water (2%)
Dark vegetation (4%)
Light vegetation (3%)
Urban and peri-urban vegetation (5%)
Empty land (10%)
Densely populated areas, >36° building orientation (8%)
Densely populated areas, <36° building orientation (34%)
Sparse-moderately populated areas (34%)
Cluster membership for 256 x 256 pixel tiles
Water (2%)
Dark dense vegetation (5%)
Light vegetation (6%)
Empty land (10%)
Densely populated areas, >36° building orientation (7%)
Densely populated areas, <36° building orientation (21%)
Buildings surrounded by vegetation (13%)
Roads and sparse-moderately populated areas (36%)
100%
75%
50%
25%
B
Cluster membership for 384 x 384 pixel tiles
Water (2%)
Dark vegetation (5%)
Light vegetation (6%)
Densely populated areas, >36° building orientation (15%)
Densely populated areas, 10-36° building orientation (9%)
Densely populated areas, <10° building orientation (15%)
Roads and sparse-moderately populated areas (30%)
Buildings surrounded by vegetation and peri-urban areas (19%)
Cluster membership for 256 x 256 pixel tiles
C
Cluster membership for 512 x 512 pixel tiles
Water (1%)
Dark vegetation (5%)
Light vegetation (12%)
Densely populated areas, >36° building orientation (9%)
Densely populated areas, 10-36° building orientation (7%)
Densely populated areas, <10° building orientation (11%)
Roads and sparse-moderately populated areas (16%)
Buildings surrounded by vegetation and peri-urban areas (38%)
Cluster membership for 256 x 256 pixel tiles

Appendix Fig. A.4: Influence of tile size on cluster membership.

The plots show the co-occurrence of assignment to specific clusters, comparing the 128 x128 pixels analysis (A) with the main analysis, 384 x 384 pixels analysis (B) with the main analysis, and 512x 512 pixels analysis (C) with the main analysis. The co-occurrence is calculated by spatially joining the 256 x 256 pixel grid with either (A), (B) or (C) and computing a frequency table of cluster membership. The values are presented as, and coloured by, percentages of the main analysis.

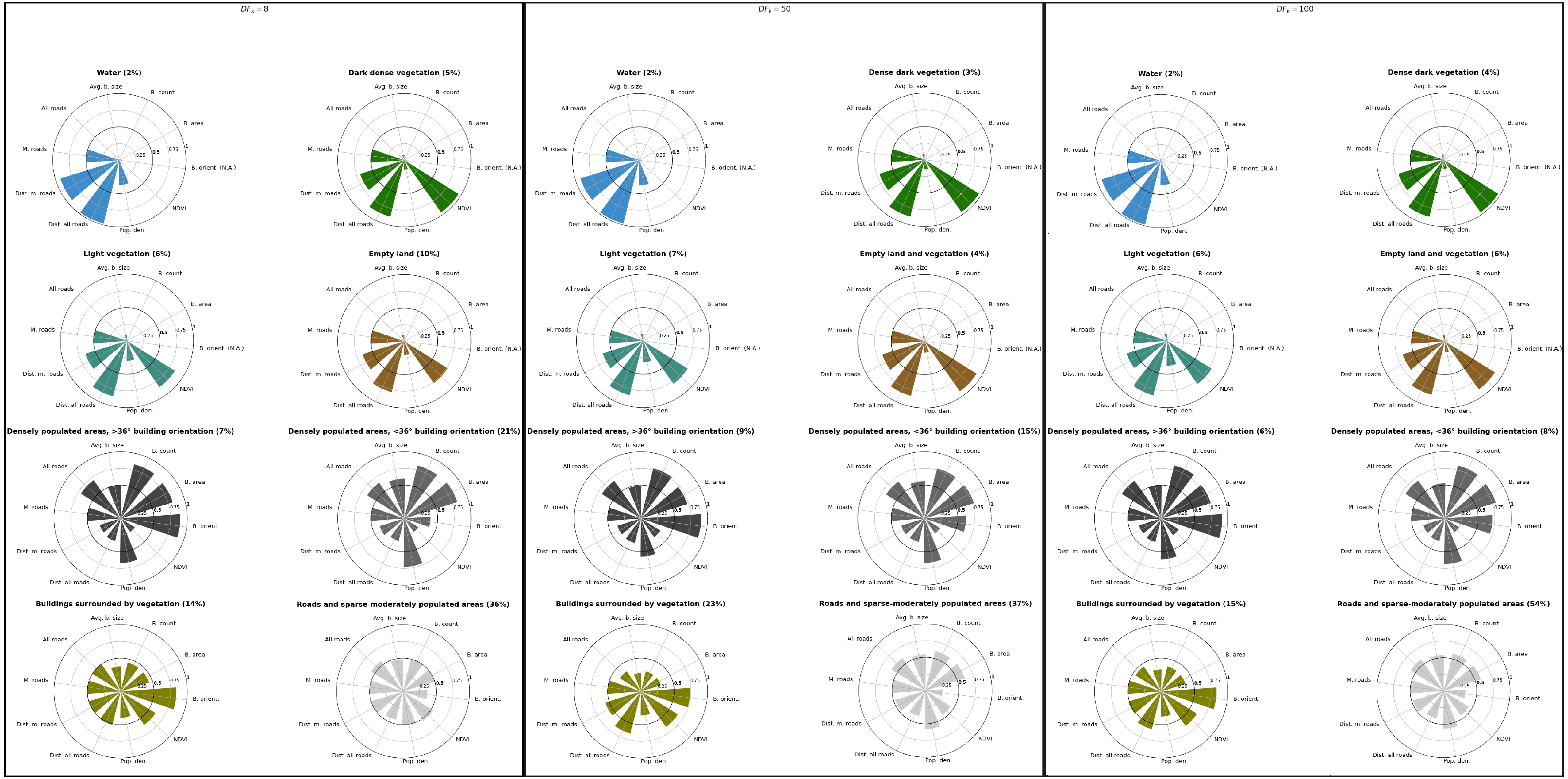

**B. count**	Building count
**B. area**	Building area
**B. orient.**	Building orientation
**NDVI**	Mean NDVI
**Pop. den.**	Population density
**Dist. all roads**	Min. distance to all roads
**Dist. m. roads**	Min. distance to major roads
**M. roads**	Length of major roads
**All roads**	Length of all roads
**Avg. b. size**	Mean building size

Appendix Fig. A.5: Impact of feature extraction hyperparameter *k* on learning of deep features (DF): DF_{k8}, DF_{k50}, and DF_{k100} (left to right).

Built and natural environment and demographic characteristics of the clustered deep features (DF_{k8}, DF_{k50}, and DF_{k100}) are shown as radar charts similar to those in Figure 3. Each environmental and demographic variable is scaled with a quantile transformer, a non-parametric transformation to map the data to a uniform distribution with values between 0 and 1 (0.5 indicating the median value of that certain variable across all tiles in the entire image). Tiles with no buildings were included in summary statistics for building count and building area (as zeros) but excluded from calculation of summary statistics for average building size and orientation so that zero is not used in the denominator.

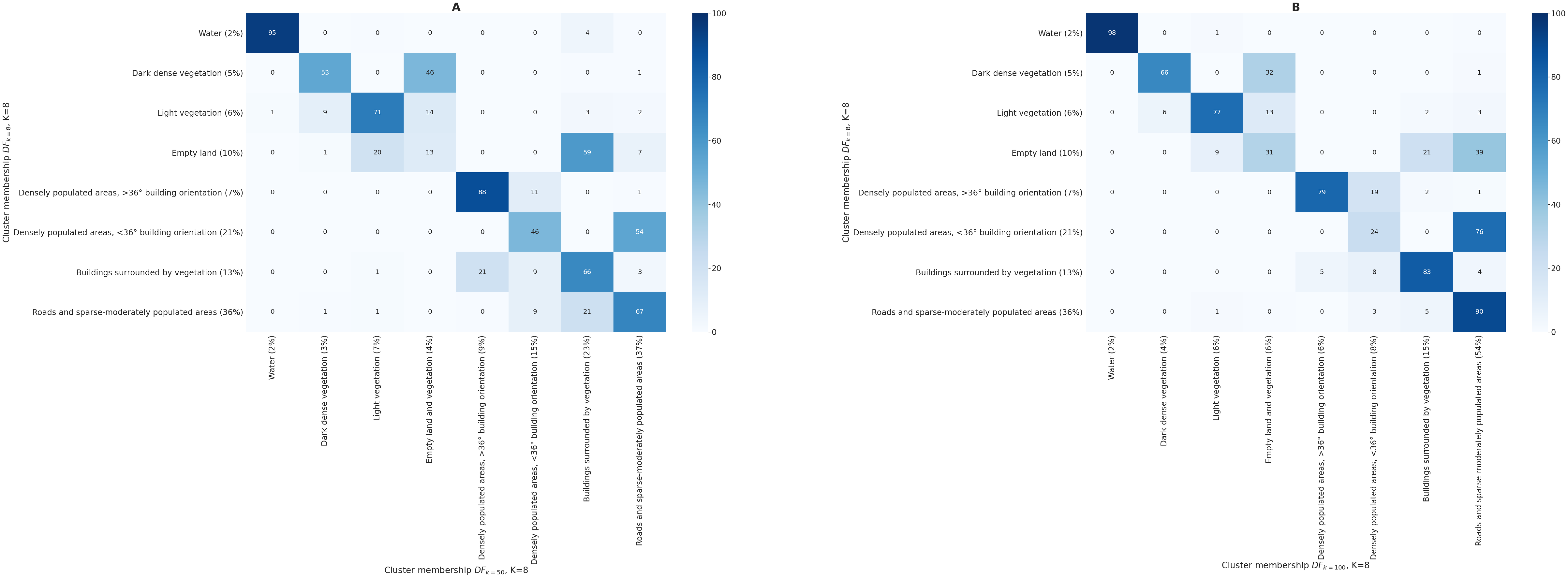
A
Water (2%) 95 0 0 0 0 0 4 0
Dark dense vegetation (5%) 0 53 0 46 0 0 0 1
Light vegetation (6%) 1 9 71 14 0 0 3 2
Empty land (10%) 0 1 20 13 0 0 59 7
Densely populated areas, >36° building orientation (7%) 0 0 0 0 88 11 0 1
Densely populated areas, <36° building orientation (21%) 0 0 0 0 0 46 0 54
Buildings surrounded by vegetation (13%) 0 0 1 0 21 9 66 3
Roads and sparse-moderately populated areas (36%) 0 1 1 0 0 9 21 67
Water (2%)
Dark dense vegetation (3%)
Light vegetation (7%)
Empty land and vegetation (4%)
Densely populated areas, >36° building orientation (9%)
Densely populated areas, <36° building orientation (15%)
Buildings surrounded by vegetation (23%)
Roads and sparse-moderately populated areas (37%)
Cluster membership $DF_{k=8}$, K=8
Cluster membership $DF_{k=50}$, K=8
0 20 40 60 80 100
B
Water (2%) 98 0 1 0 0 0 0 0
Dark dense vegetation (5%) 0 66 0 32 0 0 0 1
Light vegetation (6%) 0 6 77 13 0 0 2 3
Empty land (10%) 0 0 9 31 0 0 21 39
Densely populated areas, >36° building orientation (7%) 0 0 0 0 79 19 2 1
Densely populated areas, <36° building orientation (21%) 0 0 0 0 0 24 0 76
Buildings surrounded by vegetation (13%) 0 0 0 0 5 8 83 4
Roads and sparse-moderately populated areas (36%) 0 0 1 0 0 3 5 90
Water (2%)
Dark dense vegetation (4%)
Light vegetation (6%)
Empty land and vegetation (6%)
Densely populated areas, >36° building orientation (6%)
Densely populated areas, <36° building orientation (8%)
Buildings surrounded by vegetation (15%)
Roads and sparse-moderately populated areas (54%)
Cluster membership $DF_{k=8}$, K=8
Cluster membership $DF_{k=100}$, K=8
0 20 40 60 80 100

Appendix Fig. A.6: Influence of feature extraction hyperparameter *k* on learning of deep features (DF): DF_{k8}, DF_{k50}, and DF_{k100} (left to right).

The plots show the co-occurrence of cluster assignments, comparing the cluster membership of the main analysis (DF_{k8}) with the DF_{k50} analysis (A) and the main analysis (DF_{k8}) with the DF_{k100} analysis (B). The co-occurrence is calculated by computing a frequency table of cluster membership. The values are presented as, and coloured by, percentages of the main analysis.
